# Phosphatidylinositol 5 Phosphate (PI5P): From Behind the Scenes to the Front (Nuclear) Stage

**DOI:** 10.3390/ijms20092080

**Published:** 2019-04-27

**Authors:** Alessandro Poli, Antonio Enrico Zaurito, Shidqiyyah Abdul-Hamid, Roberta Fiume, Irene Faenza, Nullin Divecha

**Affiliations:** 1 National Institute of Molecular Genetics (INGM), 20122 Milan, Italy; zaurito@ingm.org; 2 The FIRC Institute of Molecular Oncology (IFOM), 20139 Milan, Italy; 3 School of Biological Sciences, University of Southampton, Southampton SO17 1BJ, UK; S.Abdul-Hamid@soton.ac.uk; 4 Department of Biomedical e Neuromotor Sciences (DIBINEM), University of Bologna, 40126 Bologna, Italy; roberta.fiume@unibo.it (R.F.); irene.faenza2@unibo.it (I.F.)

**Keywords:** PI5P, PIKFyve, myotubularin, PI5P4K/PIP4K, phosphatases, nucleus

## Abstract

Phosphatidylinositol (PI)-related signaling plays a pivotal role in many cellular aspects, including survival, cell proliferation, differentiation, DNA damage, and trafficking. PI is the core of a network of proteins represented by kinases, phosphatases, and lipases which are able to add, remove or hydrolyze PI, leading to different phosphoinositide products. Among the seven known phosphoinositides, phosphatidylinositol 5 phosphate (PI5P) was the last to be discovered. PI5P presence in cells is very low compared to other PIs. However, much evidence collected throughout the years has described the role of this mono-phosphoinositide in cell cycles, stress response, T-cell activation, and chromatin remodeling. Interestingly, PI5P has been found in different cellular compartments, including the nucleus. Here, we will review the nuclear role of PI5P, describing how it is synthesized and regulated, and how changes in the levels of this rare phosphoinositide can lead to different nuclear outputs.

## 1. Introduction

### 1.1. Phosphatidylinositol Signaling

Multiple cellular functions, including survival, proliferation, differentiation, DNA damage response, and gene transcription can be modulated by a specific network of kinases, phosphatases, and lipases able to modulate the lipid second messenger phosphatidylinositol [1,2,3,4,5]. Phosphatidylinositol (PI) is composed of two different modules: a hydrophilic inositol head group bound through a phosphodiester bond to a glycerol and two fatty acids tails that represent the hydrophobic part of the molecule. The fatty acids tails are prevalently represented by stearic and arachidonic acids but other acyl chains are known to be present [6]. Modifications of the inositol ring due to addition or removal of phosphate groups, together with hydrolysis of the phosphodiester bond by phospholipases C, are the most common changes leading to the production of second messengers involved in many cellular aspects. The first demonstration of lipids as second messengers was indicated by different works, which independently elucidated the process through which PI(4,5)P_2_ is cleaved by phospholipases C to diacyglycerol (DAG) and inositol 3-phosphate (IP3). These, in turn, contribute to protein kinases C (PKC) activation and calcium (Ca^2+^) release from the endoplasmic reticulum [1,2,3,4,5,7]. Subsequently, further studies have led to the discovery of many other PI-related pathways, including those involving several forms of phosphotransferases like PIKinases and PIPKinases [8] or phosphatases like phosphatase and tension homologue deleted on chromosome 10 (PTEN) and SH2-domain containing inositol phosphatase 2 (SHIP2) [9,10].

### 1.2. Nuclear Lipid Signalling: Focus on Nuclear Phosphoinositides

PI signaling was first described at the plasma membrane level, which involves PI anchored to cell membranes through DAG molecules [1,2,3,4,5]. Interestingly, it soon became clear that PIs and PI-related enzymes could be present in different cellular compartments, including cytoplasmic organellar membranes and nuclei. Nuclear PI fraction was expected to exist due to the presence of the nuclear membrane, a bilayer formed by lipids and proteins connected to the endoplasmic reticulum (ER) [11,12]. However, different reports have indicated that nuclei almost completely depleted of nuclear envelope are still characterized by the presence of many PIs and PI-related proteins like phospholipases C, phosphatidylinositol phosphate kinases (PIPKs) and diacylglycerol kinases (DGKs) [13,14,15,16,17,18,19]. Strikingly, under different stimuli, these enzymes are able to change the nuclear pool of PIs [14,16,20,21]. This evidence unequivocally demonstrated the existence of PI signaling completely localized within the nuclear compartment of cells.

Seven different phosphoderivatives of PI are known to exist and these are modulated by an intricate network of enzymes whose related pathways often intertwine. Despite the rarity of these second messengers when compared with other lipids like phosphatidylcholine or phosphatidylserine (which represent around the 12–20% of the cellular lipid pool) [1,2,3,4,5], they have been described as modulating many cellular functions acting in different cellular compartments. Among PI’s phosphoderivatives, here we will focus on phosphatidylinositol 5-phosphate (PI5P) and will particularly describe its involvement in nuclear outputs.

## 2. Phosphatidylinositol 5 Phosphate: A Rare But Essential Lipid

### 2.1. PI5P Discovery

Out of the different phosphoinositides, PI5P represents only 0.5% of the PI pool present in the cells. However, its function as a second messenger has been widely investigated. Its levels strongly fluctuate due to external stimuli such as TCR activation, insulin treatment, oxidative stress, and pathogen cellular invasion [22]. PI5P was the last PI to be discovered. In the late 1980s, experiments regarding the purification of proteins involved in PI signaling indicated the existence of two related subfamilies of phosphatidylinositol phosphate kinases (PIPK) named type I and type II PIP5K. They were thought to both be able to phosphorylate PI4P, leading to PI(4,5P)_2_ production. However, in 1997, Rameh and colleagues, studying the substrate specificity of these two PIPK classes, found that type II enzymes were able to specifically phosphorylate another lipid substrate to lead to PI(4,5)P_2_, which turned out to be PI5P. As synthetic purified lipid substrates were not available at the time, the issue with PI5P detection was due to the contamination of PI4P bovine brain preparations used for experiments involving PI5P. This delayed the detection of the real function of type II PIPK, which from that point was renamed PI5P4K/PIP4K [23] (see later).

### 2.2. Changes in PI5P Levels Regulate Many Cellular Functions

As already stated, the pool of cellular PI5P can be regulated by many external factors and stimuli. For instance, upon TCR stimulation, quick (two minute) and transient accumulation of PI5P in Uh78 cells occurs. PI5P is in turn bound by DOK proteins, leading to IL-2 promoter activity in T cells [24]. In addition, induction of platelet aggregation by thrombin treatment has been partially connected to a three-fold increase in PI5P levels in cells [25]. Other reports have described insulin treatment as able to increase levels of PI5P in 3T3-L1 adipocytes, CHO cells stably expressing insulin receptors, and skeletal muscle cells [26]. Interestingly, insulin-dependent PI5P accumulation has been connected with GLUT4 internalization-enhancing glucose uptake from the extracellular environment [27]. On the other hand, increased levels of PI5P obtained by insulin or infection by *Shigella flexeneri* PI(4,5)P_2_ 4-phosphatase IpgD lead to actin remodeling and endosome formation through TIAM1 [28]. *S. flexeneri* infection-related changes of PI5P have also been proposed to internalize and degrade cell surface levels of ICAM-1, inhibiting neutrophils recruitment [29]. Recently, other pathogen signatures like lipopolysaccharides (LPS) and viral dsRNA have been found to positively affect PI5P amounts in host cells, which have been described as being involved in toll-like receptor-related pathways [30]. All these reports showed that fluctuations of PI5P in cells can be linked to different external stimuli and signaling pathways.

## 3. Enzymes Involved in the Turnover of PI5P

**Figure 1:**
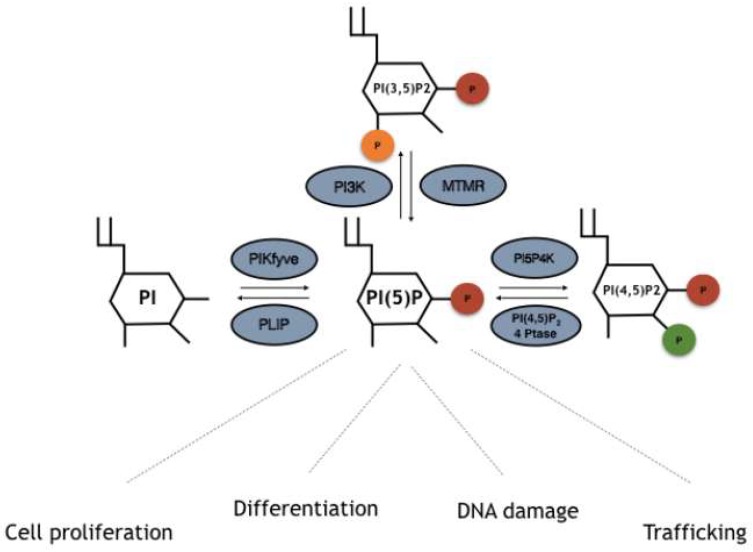
Enzymes involved in phosphatidylinositol 5 phosphate (PI5P) turnover and array of kinases and phosphatases involved in PI5P turnover. PI5P can be directly synthesized by PIKFyve phosphotransferases through direct phosphorylation of phosphatidylinositol (PI) on position 5 of the inositol ring. Moreover, MTMR phosphatases can remove a phosphate group on position 3 from PI(3,5)P_2_, leading to increased amount of PI5P levels. Finally, PI5P4K/PIP4Ks directly phosphorylate PI5P on position 4 leading to PI(4,5)P_2_ synthesis, an event that can be counterbalanced by type I/II 4-phosphatases which remove the phosphate group on position 4.

Although PI5P levels can change upon different stimuli, how this occurs is not always understood. Several pathways underlying PI5P synthesis have been described, including direct processes through phosphorylation of PI on position 5, or indirect-like de-phosphorylation of PI(3,5)P_2_ (Figure 1). Moreover, the balance between PI(4,5)P_2_ and PI5P mediated by PIP4K has also been found to be important for cellular control of the PI5P pool. Here we will describe the different pathways which lead to PI5P production in cells.

### 3.1. PIKfyve/Phosphatidylinositol-3-Phosphate 5-Kinase

PIKfyve, also known as phosphatidylinositol-3-phosphate 5-kinase type III or PIPKIII, is an established evolutionarily conserved PIK present in animals, plants and fungi. It possesses a FYVE zinc finger domain, named after the proteins in which it was first identified: Fab1p (the yeast orthologue of PIKfyve), YOTB, Vac1 (vesicle transport protein), and EEA1 (Early Endosome Antigen 1) [31,32,33,34]. This domain has a small cysteine-rich Zn_2+_ binding domain, characterized by a basic motif in the first β-strand (R/K) (R/K) HHCR which primarily allows phosphatidylinositol 3 phosphate (PI3P) binding. PIKfyve is a large protein involved in endosome processing, HIV and Salmonella replication, and type 2 diabetes, while mutations in its coding gene are connected to corneal fleck dystrophy (CFD) [34,35]. Interestingly, together with its capacity to phosphorylate PIs, it possesses protein kinase activity towards non lipid substrates [36]. Nevertheless, in vitro and in vivo evidence has described PIKfyve as able to bind and phosphorylate the lipids PI3P and PI, leading to the synthesis of PI(3,5)P_2_ and PI5P respectively [31,32,33,34] (Figure 1). Overexpression or silencing/inhibition of PIKfyve leads to changes in the levels of these two PIs. Most PI5P production in cells is thought to be due to PIKfyve activity via both direct and indirect pathways. Indeed, as stated, PIKfyve is able to directly phosphorylate PI rings in position 5, leading to synthesis of PI5P (the direct pathway) [31,32,33,34,37]. Another proposed model of PI5P synthesis is related to dephosphorylation of PIKfyve-derived PI(3,5)P_2_ by 3-phosphatases named myotubularins (the indirect pathway, see next) [38,39].

### 3.2. MTM-MTMR/Myotubularins

Myotubularin 3-phosphatases represent a family of proteins conserved in eukaryotes and composed of 15 members named MTM1 and MTMR1–14 [40,41,42,43]. These enzymes share a structural motif which is represented by a PH-GRAM (pleckstrin homology-glucosyltransferases, rab-like GTPase activators, and myotubularin), catalytic protein tyrosine phosphatases (PTP) domains, and a coiled-coil motif. Some of the isoforms also contain FYVE-, PH-, and PDZ-binding sites [40,41,42,43]. The active site of the protein is represented by a Cys-X_5_-Arg motif, which allows hydrolyzation of phosphodiester bonds on a cysteine nucleophile and an arginine residue, binding an oxygen atom onto the phosphate groups [40,41,42,43]. These catalytic residues can be altered by missense substitutions in several isoforms. This divides myotubularins into active (MTM1, MTMR1–4, MTMR6, MTMR7–8, and MTMR14) and inactive (MTMR5 and MTMR9–13) phosphatases [44,45]. Throughout the years, MTMs have been described as being able to bind and dephosphorylate PI3P and PI(3,5)P_2_ to PI and PI5P, respectively. Reports on the crystal structure of MTMR2 have unraveled the molecular basis of PI3P and PI(3,5)P_2_ binding through its PH-GRAM domain [45,46,47] (Figure 1). Finally, this class of PI phosphatases is known to play a role in endocytosis and membrane trafficking, cell proliferation, differentiation, and cell junction dynamics. Mutations on the genes encoding myotubularin enzymes have been found in neuromuscular diseases or have been associated with metabolic syndromes, obesity, and cancer.

### 3.3. Phosphatidylinositol 5 Phosphate 4 Kinases (PI5P4K/PIP4K)

Phosphatidylinositol 5 phosphate 4 kinases, or type II PIPKs, represent a family of enzymes able to phosphorylate PI5P in order to produce PI(4,5)P_2_ [23]. PIP4Ks are conserved in different species spanning from flies and worms to mice and humans. Mammalians are characterized by the presence of three different isoforms, namely, PIP4K2A, PIP4K2B, and PIP4K2C [4,8,48,49,50]. They share dimerization and lipid kinase domains and carry main differences in amino acid sequences found at -N and -C termini, which confer each isoform specific characteristics [4,8,49,50,51]. PIP4K2A is considered the most active isozyme if compared to PIP4K2B, while PIP4K2C possesses a limited capacity to phosphorylate PI5P [4,8,48,49,50]. Interestingly, PIP4K2B preferentially uses GTP instead of ATP for its kinase function [52,53]. PIP4K isozymes also differ from each other for their intracellular localization: 2A is mostly located in the cytoplasm/membrane, 2B can also be found in the nucleus, and 2C is found in not well defined membraneless compartments. As already indicated, this class of enzymes was first discovered in 1997 by members of Cantley’s lab, who were able to overcome an issue related to the mix between PI4P and PI5P in bovine brain preparations [23]. This finding rendered the study of PIP4K substrate specificity possible and described those proteins as different from their related family of PIP5K [54]. In any case, the capacity of PIP4K to produce PI(4,5)P_2_ is considered minor with respect to PIP5K, so they are suggested as being involved in the regulation of PI5P levels in cells [4,8,49,50] (Figure 1). Indeed, knockdown/inhibition of PIP4Ks in mammalian cells or knockout in drosophila leads to increased levels of PI5P, with almost no effects on the pool of PI(4,5)P_2_ [49,55,56]. These proteins have been recently connected with many cellular functions, including DNA damage, cell proliferation, and chromatin remodeling, and have been proposed as possible targets for treatment of cancer or autoimmune diseases [57,58,59,60].

### 3.4. Type I/II PI(4,5)P_2_ 4-Phosphatases

Type I/II PI(4,5)P_2_ 4-phosphatases are human phosphatases able to specifically target PI(4,5)P_2_ and hydrolyze the phosphodiester bond of D4 phosphate on the inositol ring, leading to synthesis of PI5P in human cells. Type I/II 4-phosphatases share a Cys-X_5_-Arg motif with the *Shigella flexenery* PI(4,5)P_2_ 4-phosphatase IpgD. This, once injected or expressed into host cells, leads to a specific and strong increase in PI5P levels through PI(4,5)P_2_ hydrolysis [61,62,63] (Figure 1). Mammalian type I/II PI(4,5)P_2_ 4-phosphatases were described for the first time by Ungewickell et al. and were reported as being located in endosomal/lysosomal membranes in epithelial cells. In addition, upon cellular stress, type I 4-phosphatase can localize in the nucleus, where it regulates p53 dependent apoptosis [64].

## 4. PI5P and Nuclear Outputs

The role of PI5P as a second messenger has been widely investigated. In particular, many processes regulated by changes in PI5P levels have been described in relation to the nuclei. This began with evidence collected during the study of the cell cycle of murine erythroleukemia (MEL) cells, which showed a strong increase of the nuclear PI5P pool during G1/S transition [14,65]. This led to the first ideas about possible roles of this phosphoinositide in the regulation of nuclear processes [66]. In fact, throughout the years, it has turned out that PI5P is involved in many nuclear outputs such as chromatin remodeling, gene expression, or responses to stressors like UV irradiation or genotoxic factors. Here, the nuclear role of this monophosphoinositide will be reviewed by considering and describing the main published works so far.

### 4.1. Nuclear PI5P Regulates ING2-Mediated p53 Acetylation and Apoptosis During Stress Response

**Figure 2:**
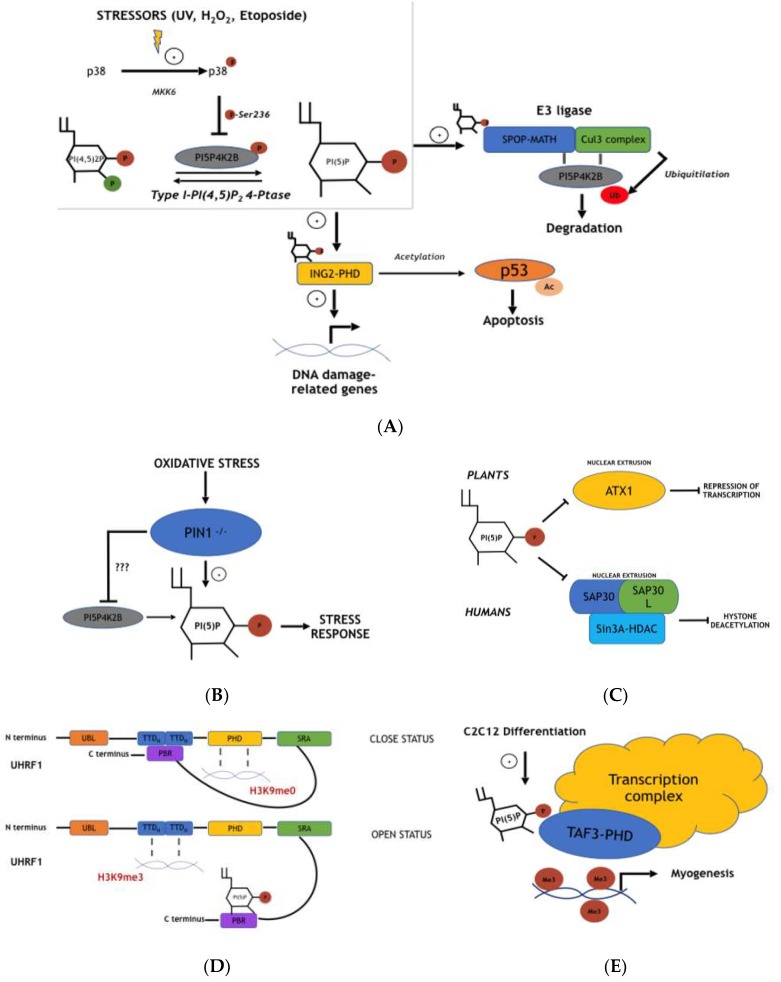
The role of PI5P in nuclear outputs and PI5P as lipid second messenger involved in different nuclear functions. (**A**) Increased levels of PI5P during cellular stress affect ING2 and cellular responses through PIP4K2B and type I PI(4,5)P_2_ 4-ptase activity. Moreover, PI5P can modulate Cul3 E3 ligase/substrate-specific adaptor protein (SPOP) binding, leading to PI5P4K2B/PIP4K2B degradation. (**B**) Pin1 regulates PI5P upon oxidative stress. (**C**) ATX1 and SAP30/SAP30L are extruded from the cell nucleus by PI5P binding. (**D**) Uhrf1 conformation and binding capacity of either H3K9me3 or H3K9me0 DNA regions is mediated by interaction with PI5P. (**E**) TAF3-mediated regulation of genes involved in myogenic differentiation requires PI5P.

The first evidence of the major role of PI5P in nuclei was found by Gozani et al. in 2003 (Figure 2A) [67,68,69]. Here, the authors demonstrated that ING2 could bind mono-phosphoinositides, particularly PI5P. Inhibitor of growth (ING) enzymes represent a family of proteins characterized by five members, ING1–5, which are able to associate to histone acetyltransferases (HAT), histone deacetylases (HDAC), and acetyl-transferase complexes, and are involved in cell survival, cell cycle, and apoptosis [67,68,69]. For these reasons, INGs are considered to be either oncogenes or oncosuppressors, and their expression has been found to be down regulated in many tumor types. Indeed, ING1–5 expression is positively involved in p53-related apoptosis. INGs can lead to enhanced acetylation of p53 both directly (p300 and HAT activity) or indirectly (NAD-dependent deacetylase sirtuin-1/SIRT1 inhibition) by increasing its activity [67,68,69]. Every ING isoform shares an identical N-terminal domain and a very similar C-terminal part. The latter is characterized by the presence of nuclear localization signal sequences and by a plant homeo domain (PHD) finger, a Cys_4_-His-Cys_3_ motif present in enzymes involved in chromatin regulation. ING2 was identified as a PI5P binding protein using different approaches, including PtdInsP-affinity resin, protein-lipid blot assay, and surface plasmon resonance (SPR) [70]. Notably, it is able to bind slightly to PI3P and PI(3,5)P_2_. In particular, the PHD zinc finger motif has been described as indispensable for PI binding. Indeed, a zinc chelator, TPEN [71], has been shown to be able to decrease PI5P-ING2 interaction. Moreover, NMR solution analyses of the PHD finger structure revealed three lysine residues (K49, K51, and K53) as part of a basic stretch on the protein surface involved in the binding of PI5P. The PHD finger presence in different enzymes was then described as being involved in different PI bindings. Interestingly, strong PI5P induction at the plasma membrane, due to expression of the bacterial protein IpgD, was shown to strongly recruit ING2 out of the nucleus. This was confirmed by overexpression of PIP4K2B, able to modulate PI5P levels in the nuclei, which was sufficiently able to alter the ING2-association with the chromatin/nuclear matrix. Furthermore, as already stated, ING2 is involved in p53 acetylation and related apoptosis. Authors, then, have demonstrated that only overexpression of wild type ING2 can lead to effects on p53 acetylation status and cell viability, while ING2 mutants, lacking PIP binding sites, are not able to affect this process. In addition, induction of p53 acetylation, increases in p21 levels, and apoptosis due to etoposide treatment are inhibited when the isolated PHD finger of ING2 is expressed. As this domain can bind PI5P, it acts as a dominant negative factor in ING2-p53 mediated cell death. Altogether, the data summarized here described PI5P as a pivotal player in ING2 function and localization. Nevertheless, how and why PI5P levels change upon cellular stress and/or if ING2 is able to sense this phenomenon were not completely demonstrated.

### 4.2. PIP4K2B and Type I PI(4,5)P_2_ 4-Phosphatase Signaling Pathways Control Nuclear PI5P Levels and ING2 Functions During Cellular Stress Response

Two different explanations involving both PIP4K2B and type I PI(4,5)P_2_ phosphatase for the control of ING2-p53-related acetylation and cell death, have been proposed (64,72). Jones et al. demonstrated that UV irradiation, oxidative stress (through hydrogen peroxide (H_2_O_2_) or l-buthionine[S,R]-sulfoximine), or etoposide treatment can specifically induce accumulation of PI5P in the nuclei of MEL cells, particularly in the chromatin-enriched fraction (CEF) [72] (Figure 2A). This phenomenon was shown to be due to inhibition of PIP4K2B activity, as nuclear 3-phosphatases were not affected. Immuno-precipitation of PIP4K2B and a subsequent kinase activity assay, performed in normal or UV irradiation treated MEL cells, confirmed impaired phosphorylation of PI5P by PIP4K2B under the second condition. Interestingly, the effects of UV on PIP4K2B were found to be reduced, blocking p38 kinase (a member of the mitogen-activated protein kinases (MAPKs) family) [73]. Indeed, only the overexpression of the constitutively activated MKK6 (MKK6+), an up-stream p38 activator, and not of MKK62, the homologous kinase dead mutant, leads to the inhibited function of PIP4K2B in vitro. In addition, mass spectrometry revealed that p38 was able to phosphorylate PIP4K2B on Ser326 upon UV irradiation. As a consequence of lost PIP4K2B activity, PI5P nuclear levels increased in MEL cells. This evidence was supported by RNA interference with PIP4K2B, which phenocopied the effects on the nuclear PI5P pool during UV irradiation. Finally, as ING2 localization was already connected to PI5P, the authors showed that modulation of PIP4K2B levels in MEL cells upon UV irradiation or oxidative stress can change the CEF accumulation of ING2 through a PI5P dependent pathway. Indeed, stressor treatment leads to the activation of p38 and subsequent phosphorylation of PIP4K2B on Ser326; this diminishes the enzyme activity, increasing PI5P levels in the nuclei of the cells that, in turn, function as an attractor for ING2. On the other hand, a different but related signaling pathway has been described by Zou et al. [64]. They reported the role type I PI(4,5)P_2_ 4-phosphatase plays in stress-regulated apoptosis. Inducible overexpression of type I 4-phosphatase is sufficient to increase PI5P levels through dephosphorylation of PI(4,5)P_2_ in Hek293 cells, while type II does not affect the PI5P pool (Figure 2A). As ING2-related acetylation of p53 increases its stability and function, the authors showed that silencing or overexpression of type I PI(4,5)P_2_ 4-phosphatase in cells treated with the genotoxic compound etoposide leads to altered p53 protein levels. In particular, overexpression of type I phosphatase causes a definite increase in p53 acetylation and, as a consequence, cell death. This effect is partially inhibited by overexpression of PIP4K2B, since it has been previously described as regulating ING2-dependent p53 acetylation. Furthermore, the authors demonstrated that type I 4-phosphatase signaling is connected to p53 acetylation through ING2. Indeed, knockdown of ING2 together with overexpression of type I 4-phosphatase shows decreased p53 acetylation levels, indicating ING2 as part of the process. In addition, upon cellular stress, type I 4-phosphatase, usually located in the cytosol in homeostatic conditions, partially translocates inside the nucleus. Here, it was described as controlling the levels of PI5P. This evidence indicates the role of nuclear PI5P in the control of cell apoptosis through ING2-dependent acetylation of p53, a process regulated by both PIP4K2B and type I 4-phosphatases, which act on PI5P and PI(4,5)P_2_, respectively.

### 4.3. PI5P Involvement in ING2 DNA Binding and Stability

Although PI5P involvement in ING2 function has been widely described, a recent report published by Bua et al. has been necessary to better explain how PI5P modulates ING2-dependent transcription during cellular stress response processes [74] (Figure 2A). As PHD fingers allow binding with DNA and/or phosphoinositides, the authors expressed, in cell lines, different ING2 isozymes whose PHD fingers are mutated. Those mutants are able to interact only with H3K4me3 and neither PI5P (ING2_6k/Rmt_) nor the two of them (ING2_D-6k/Rmt_). This allowed the authors to study the importance of ING2-PI5P interaction in terms of the ING2-related gene expression program during stress response. Genome-wide promoter occupancy and gene expression microarray analyses showed ING2 to specifically bind different promoter regions of genes involved in cell cycle and chromosome reorganization upon etoposide treatment. Interestingly, ING2 negative regulation of this cohort of genes is strongly impaired by the expression of the ING2_6k/Rmt_ mutant (no PI5P bound). This was confirmed by changing the nuclear PI5P amount through PIP4K2B expression. In fact, lower nuclear PI5P levels decrease the capacity of ING2 to bind to promoter regions of specific target genes, thus inhibiting its suppressive activity. This evidence sheds new light on the meaning of PI5P for ING2 function, indicating this phosphoinositide as fundamental to ING2-DNA binding during stress response.

### 4.4. Pin1-Dependent PI5P Modulation Affects Oxidative Stress Cellular Response and Reactive Oxygen Species (ROS) Production

Peptidyl-prolyl isomerase (PPIase) Pin1 is an enzyme which is able to catalyze proline cis-trans isomerization affecting protein folding and conformation. It has been proposed as a novel player for PI5P generation upon stress (oxidative) response [75,76,77] (Figure 2B). Pin1 is characterized by an N-terminal WW-domain which allows binding to phosphorylated serine and threonine residues close to a proline residue on target proteins, and a C-terminal PPIase catalytic domain. A study by Keune et al. showed that this protein is able to bind both PIP4K2A and PIP4K2B, but not PIP4K2C [75]. Affinity purification of GST-Pin1 with a general lipid kinase substrate (Folch lipid fraction from brain supplementation of PI5P) allows for the isolation of enzymes able to produce PIP_2_, which have turned out to be PIP4K2A and PIP4K2B. As previously stated, Pin1 is able to bind Ser/Thr phosphorylated proteins through its WW-domain. Moreover, PIP4K2B had been known to be phosphorylated upon stress on Thr322 (unknown effector) and on Ser326 by p38. This suggested that these two residues could be fundamental to the interaction between Pin1 and PIP4K2B. In fact, experiments of protein binding employing PIP4K2B mutated on these two amino acids, as well as mutant WW-Pin1 (no protein binding), have confirmed this. In particular, the concomitant mutation of both Thr322 and Ser326 abolishes the interaction of the two enzymes. On the other hand, UV irradiation or MKK6+ expression are able to enhance this interaction, indicating p38-dependent Ser326 phosphorylation to be important for the entire process. As PIP4K2A conserves the same Ser326 residue, together with more than 80% of homology with 2B isoform, it has been suggested that p38-related phosphorylation could affect it as well. Next, lipid kinase activity of PIP4Ks was investigated. This was performed through functional assays of the purified enzymes using GST-fused WT, WW, or PPI (kinase dead protein) mutants of Pin1. The amount of lipid kinase activity was highly reduced with WT Pin1 when compared to PPI mutants, indicating the role of Pin1 in the inhibition of PIP4Ks. Moreover, PIP4K2B and Pin1 were both found to localize in the nuclei of HeLa cells. This suggests that the control of PIP4K2B function by Pin1 can lead to changes in nuclear PI5P levels. However, Pin1 -/- MEL cells generate high levels of PI5P upon H_2_O_2_ treatment and show more resistance to stress compared to their WT counterparts. This is partially contradictory, considering the suggested role of Pin1 in the negative modulation of PIP4K activity. Reintroduction of Pin1 in -/- cells or overexpression of PIP4K2A reduces the levels of PI5P upon H_2_O_2_ treatment and decreases cell viability, supporting the notion of PI5P as strongly connected with stress response processes. Finally, PI5P changes upon oxidative stress have been reported to regulate ROS accumulation. Indeed, overexpression of PIP4K2A in Pin1 -/- MEL cells treated with H_2_O_2_, and subsequent down regulation of PI5P levels, reduces the expression of NRF2 transcription factor related genes like NQO1 and GSTA1. These are known to be involved in detoxification by ROS accumulation [78]. Data summarized here indicates the role for PI5P in ROS accumulation and cell resistance to oxidative stress.

### 4.5. Cul3 E3 Ligase Complex Activity Is Inhibited by PI5P through Inhibition of Substrate-Specific Adaptor Protein (SPOP)

Another report investigating p38/PIP4K signaling revealed this pathway to be involved in SPOP function [79] (Figure 2A). Speckle-type pox virus and zinc finger protein (POZ) is an E3 ubiquitin ligase adaptor protein which is involved in substrate recruitment of the E3 ubiquitin ligase complex Cullin 3 (Cul3)-RING3 [80]. This consists of a molecular scaffold protein, Cul3, being able to connect SPOP to the catalytic part, represented by the RING finger domain and E2 ubiquitin conjugating enzyme. SPOP is able to bind the Cul3 complex via a broad complex/Tramtrack/bric-a-brac (BTB) domain and specific protein targets via a meprin and TRAF-C homology (MATH) domain. Bunce et al. have demonstrated that PI5P can bind SPOP, affecting the function of the complex [81]. The authors identified SPOP as a binding-partner of PIP4K2B through a yeast two-hybrid screen, which was confirmed by different binding-assays both in vitro and in vivo. In particular, PIP4K2B was found to interact with MATH and not with the BTB domain, and to co-localise in nuclear speckles with SPOP. Moreover, ubiquitylation assays showed that SPOP promotes Cul3-based ubiquitin ligase related PIP4K2B ubiquitylation and degradation. This process has been linked to p38 MAPK signaling. In fact, co-overexpression of MKK6+ (constitutively active p38-activating kinase), together with Cul3 and SPOP, leads to an even stronger degradation of PIP4K2B, an event found to be independent of Ser236 phosphorylation of PIP4K2B by p38 MAPK. Strikingly, Cul3-SPOP complex activation is positively modulated by PI5P. Indeed, overexpression of a kinase inactive PIP4K2B or type I/II 4-phosphatases leads to increased levels of PI5P in Hek293 cells. This is followed by degradation of PIP4K2B, together with well-characterized targets of the Cul3 E3 ligase complex, including Daxx and Pdx1 (used as positive controls in these experiments). Evidence collected here sheds light on a new pathway involving p38-related PIP4K2B degradation and PI5P regulation upon stress response.

### 4.6. ATX1 Nuclear Export and Function Is Negatively Associated with Nuclear PI5P Pool Changes

Nuclear changes of PI5P levels have also been connected to gene transcription and chromatin remodeling through the regulation of ATX1 in plants [82] (Figure 2C). Arabidopsis trithorax (*atx*) genes encode plant methyltransferase proteins, which are able to methylate lysine residues on the tail of H3 histones and are thus involved in chromatin remodeling and gene expression [83]. The *atx* genes are the plant homologues of the mammalian Trithorax Group (TrxG) of proteins and share an evolutionary-conserved domain named suppressor of variegation, enhancer of zeste, and trithorax (SET) which is fundamental for lysine methylation of histone 3. The *Arabidopsis* genome is characterized by the presence of 49 genes encoding for proteins which possess a SET domain, named the Set Domain Group (SDG), and is divided into five distinct classes [84]. Among the class III genes, ATX1 contains a SET domain which is involved in H3K4 trimethylation. Surprisingly, mutations on the *atx1* gene do not totally impair this process, as about 85% of H3K4me3 is maintained in the ATX1 mutant *Arabidopsis* [82]. This suggests that ATX1 is part of a more complex set of histone tail modification proteins. In addition, ATX1 has been described as having pleiotropic functions. In fact, it is involved in the regulation of genes encoding the organ development of flowers (i.e., petals and stamens) and responses to external stressors [82]. Whole genome analyses of ATX1-mutated plants at the bolting stage revealed changes in about 12% of the genes already expressed at that specific developmental step, especially genes involved in physiological processes and in response to stimuli. Analyses of the structure of ATX1—in particular its PHD finger sequence—have revealed the presence of an enriched basic motif able to bind PIs, which have turned out to be able to specifically bind to PI5P both in vitro and in vivo. Moreover, wide genome analyses of regulated genes in ATX1 mutants and during exogenous PI5P addition revealed the negative role of the lipid in the control of its function. This is due to the nuclear extrusion of ATX1 during PI5P treatment, an event also confirmed in a subsequent report by Ndamukong et al. [85]. Here, the authors set up a dehydration-stress protocol through air-exposure of rosette leaves which led to a strong increment in PI5P (Figure 2C). Interestingly, they found that upon dehydration, a plant myotubularin homologue encoded by the *At3g10550* gene, AtMTM11, can be the main enzyme involved in PI5P production. Indeed, overexpression or depletion of AtMTM11 leads to strong changes in PI5P levels during stress. In addition, in order to investigate the reduced activity of ATX1 during stress-induced PI5P increase, *WRKY70* gene expression was analysed. In fact, ATX1 controls the methylation status of H3K4me3 at the promoter region of *WRKY70*. Regulation of PI5P levels by drought stress and AtMTM11 function indicated that ATX1 dependent transcription of *WRKY70* is strongly regulated by changes in PI5P. Moreover, increase in PI5P leads again to nuclear export and cytoplasmic localization of ATX1, a phenomenon which is related to its PHD finger and, then, to PI5P binding. Altogether, these reports described ATX1-PI5P binding as being able to modify ATX1 function and gene expression in plants.

### 4.7. SAP30/SAP30L-Sin3A-HDAC-Mediated DNA De-Acetylation Is Negatively Correlated with PI5P Nuclear Levels

As has already been reported, histone modifications are commonly used hallmarks for transcription activation or repression [86,87]. Among different the histones statuses, recruitment of histone deacetylases (HDACs) to chromatin, which leads to histone de-acetylation, is usually linked to repression of gene transcription [86,88,89]. HDACs can be moved to histones both directly (by protein-like YY1 transcription factor or retinoblastoma protein pRb) or indirectly (by protein complexes characterized by more subunits like the Mad-Max complex) [89]. Sin3A is an enzyme present in a co-repressor complex involved in HDAC DNA binding [90] that lacks the ability to directly bind DNA. It functions as a protein bridge for the formation of a repressor pool of isozymes composed of several subunits such as histone-binding proteins RbAp46 and RbAp48, RBP1, SAP130, BRMS1, SDS3, SAP30/SAP30L, and SAP18. SAP30 and SAP30L (SAP30-like) share 70% of homology and are thought to stabilize the molecules involved in the repressor complex [91]. Both these enzymes have been found in nucleoli and have been described as able to bind mono-phosphoinositides, especially PI5P [91] (Figure 2C). Multidisciplinary analyses including electrophoretic mobility shift assays (EMSA), protein purification, and protein binding microarray (PBM) have indicated that both SAP30 and SAP30L can bind DNA through a zinc-binding C2CH motif, a closely located polybasic region (PBR) in the NLS, and an intervening hydrophobic pocket. DNA binding by SAP30 and SAP30L also leads to DNA bending, as shown by circularization of short (~150 bp) DNA fragments in the presence of T4 DNA ligase. Both these enzymes have been described as binding PIs through the PBR together with the zinc finger motif. This interaction results in the necessity of modulating SAP30/SAP30L chromatin binding, leading to their cytoplasmic translocation and decreased transcription repression activity. Interestingly, these data indicate overlap of sequences able to bind PIs and DNA, leading to competitive effects which trigger DNA detachment of SAP30/SAP30L enzymes.

### 4.8. PI5P Drives Uhrf1 to Specifically Bind Either H3K9me0 or H3K9me3

Among DNA modifications, DNA and histone methylations regulate many processes, including DNA repair, transcription, and replication [86,87]. DNA methylation particularly occurs on CpG sites and is maintained through daughter cells after mitosis. In fact, DNA methyltransferasis 1 (DNMT1) is recruited to new and hemi-methylated DNA products in order to mimic CpG methylation in the new replicated DNA [92]. Ubiquitin-like, containing PHD and RING finger domains 1 (Uhrf1), an E3 ubiquitin ligase, plays a pivotal role in DNMT1 DNA docking. Uhrf1 is a protein characterized by multiple domains. The N-terminal ubiquitin-like domain (UBL) domain is necessary for the E3-ligase RING domain dependent ubiquitylation at lysine 23 of histone H3 (H23KUb), the docking site for DNMT1 DNA binding [93,94]. Moreover, the SRA domain (SET and RING finger associated) is involved in the recognition of hemi-methylated DNA sequences. On the other hand, both plant homeo domain (PHD) and tandem tudor domain (TTD) domains act as DNA “readers”, leading Uhrf1 to bind the N-terminal sequence of H3K9me0 or H3K9me3, respectively [95]. Uhrf1 can interact with DNMT1 through SRA and PHD domains [93,94]. Despite the fact that all the domains act together in the modulation of Uhrf1 DNA reading and binding, no clear evidence has demonstrated putative regulators that could drive it to preferentially bind to differentially methylated histones. Recently, Gelato et al. proposed PI5P as a fundamental actor in this process [96] (Figure 2D). In experiments performed to dissect the binding specificity of PHD and TTD domains to differentially methylated histone 3, they demonstrated that Uhrf1 can preferentially bind H3K9me3 in the presence of nuclear extracts (NE) through the TTD domain zz. On the contrary, no such nuclear extracts are required for H3K9me0 binding through the PHD domain. Further analyses of Uhrf1 have indicated the presence of a poly basic region (PBR) at the C-terminus of the protein which results in the fundamentally different DNA interaction of Uhrf1. PBR indeed is able to connect with the TTD domain, inhibiting its DNA docking capacity and leaving only the PHD domain as the main DNA targeting module. This leads to the specific “closed” status of the protein. In the presence of NE, however, the binding between PBR and TTD is broken and a rearrangement of Uhrf1 drives the enzyme to an “open” conformation with the TTD domain now able to link H3K9me3. Strikingly, a close investigation of the PBR sequence has enlightened a motif (K/R-(X)_n=3–7_-K-X-K/R-K/R) known to be possibly implicated in PI binding. Lipid dot blot experiments have demonstrated the high affinity of Uhrf1 for PI5P, although other PIs can interact slightly with it. In particular, PI5P alterations due to overexpression of PIP4K isoforms 2A and 2B are sufficient to drive Uhrf1 to specifically bind either H3K9me0 or H3K9me3. This is due to PI5P-PBR-TTD regulation. These data have evidenced a new specific role of the second messenger PI5P in the regulation of DNA methylation. In fact, PI5P is able to result in the controlling of the DNMT1-Uhrf1 interaction with specific zones of DNA either enriched in N-terminal-modified histones or not.

### 4.9. PI5P Regulates Myogenic Differentiation Modulating TAF3 DNA Binding to Specific DNA Regions

The pre-initiation complex (PIC) is a complex composed of numerous proteins (a total mass of around 800 kDa) involved in the regulation of RNA polymerase II positioning within specific DNA regions, and their subsequent transcription [97,98]. PIC formation requires the general initiation factor (GTF) TFIID, which is composed of a TATA-binding polypeptide (TBP) and TBP-associated factors (TAFs) [97,98,99]. The binding of TFIID to the TATA box at the gene promoter regions starts the recruitment of other main factors involved in RNA Pol II dependent transcription. Among the TAF proteins, TAF3 can regulate gene transcription as either part of the canonical TFIID PIC involving TBPs for promoter binding, or as part of non-canonical complexes interacting with TBP-related factor TRF3 [97,98,99]. TAF3 is indeed able to bind H3K4me3 through a PHD finger domain, and this interaction modulates TFIID recruitment/stabilization to TATA-less promoter regions. Moreover, TAF3 is characterized by tissue specific functions, including muscle differentiation [97,98,99]. Bultsma et al. proposed the role of PI5P in the regulation of myogenic differentiation through the control of TAF3 DNA binding [100] (Figure 2E). As already stated, nuclear PI5P levels can be controlled by the activity of PIP4K2B, which is highly expressed in muscle tissues [101]. PIP4K2B localization during differentiation of C2C12 myoblasts in myotubes changes, leading to enzyme nuclear exclusion after four days. This event correlates with increased nuclear PI5P throughout the process. In addition, depletion of PIP4K2B augments the nuclear PI5P pool, leading to an enhanced transcription of early and late myogenic markers including MYOG, MYH, myosin light chain (MYL), and muscle creatine kinase (CKM) [101]. Among the different PHD finger containing proteins able to interact with PIs, TAF3 is able to bind mono-phosphoinositides PI3P, PI4P, and PI5P through a PBR located in the C terminus of the PHD finger domain. Mutational changes on the PHD finger sequence allowed the authors to create different mutants of the protein which are able to only bind H3K4me3 (KK) or PI5P (DW). These, once expressed in differentiating C2C12 cells, differentially regulate genes involved in myogenic differentiation, compared to wild-type (WT) TAF3. In fact, the latter leads to high levels of MYOG, MYH, and MYL, whose expression increases even further upon PIP4K2B depletion. On the other hand, KK-TAF3-expressing cells have shown attenuated expression levels of MYOG, MYH, and MYL, which are not affected by knockdown of PIP4K2B. Transcriptomic analyses have suggested that nuclear changes in PI levels, mediated by PIP4K2B, can modulate specific genes involved in myogenic differentiation. Authors have shown that this is due to the interaction between TAF3 and PI5P. In fact, in vitro experiments of fluorescence polarization have shown that TAF3 binding of H3K4me3 is helped by PIP4K2B silencing, and, thus, by PI5P increase. These findings have also been confirmed in vivo through chip-analyses. This has allowed the assessment of specific promoter regions bound by TAF3 during C2C12 differentiation. As expected, TAF3 is enriched in PIP4K2B-regulated genes like MYOG and MYOD. Strikingly, PI binding has been found necessary for the “right” and specific TAF3 occupancy of DNA. Indeed, mutant KK-TAF3 localises in many genomic regions in a non-specific way due to the lack of PI binding. On the contrary, WT-TAF3, able to interact with PI5P, has been found in promoter regions of genes involved in differentiation of C2C12. Finally, a zebrafish model (*Danio rerio*) has been exploited to further characterise the importance of TAF3-PI5P in myogenic differentiation. Severe impairment of myofibril alignment and organization of myosin filaments have been found upon TAF3 silencing. These defects are diminished by the overexpression of human WT-TAF3 and not by KK-TAF3 or DW-TAF3 mutants. Altogether, the data described here indicates the role of nuclear PIP4K2B-PI5P in the modulation of TAF3 DNA binding and that subsequent regulation of genes involves myogenic differentiation.

## 5. Conclusions

Throughout this review we have discussed the involvement of PI5P in the regulation of several cellular functions, focusing our attention on the nuclear dynamics modulated by changes in the levels of this lipid second messenger. Specifically, we have described how different enzymes involved in the production and turnover of PI5P, comprising myotubularins, PIKFyve, PIP4Ks, and type I/II 4-phosphatases, can affect several nuclear outputs including transcription, DNA damage response, and cell differentiation. However, the study of PI5P as a second messenger is still quite challenging. This is due to a lack of specific antibodies or ways to detect it, i.e., more sensitive cellular probes, as well as methods to specifically and singularly alter its levels. In addition, other PI5P-interacting enzymes exist, but these need further validation and analysis. For all these reasons, we are sure that many new chapters in the story of this rare and little understood phosphoinositide will be written in future.
